# Trauma-focused cognitive behaviour therapy versus treatment as usual for post traumatic stress disorder (PTSD) in young children aged 3 to 8 years: study protocol for a randomised controlled trial

**DOI:** 10.1186/s13063-015-0632-2

**Published:** 2015-03-25

**Authors:** Tim Dalgleish, Benjamin Goodall, Isobel Chadwick, Aliza Werner-Seidler, Anna McKinnon, Nicola Morant, Susanne Schweizer, Inderpal Panesar, Ayla Humphrey, Peter Watson, Louise Lafortune, Patrick Smith, Richard Meiser-Stedman

**Affiliations:** Medical Research Council Cognition and Brain Sciences Unit, Cambridge, UK; Cambridgeshire and Peterborough NHS Foundation Trust, Cambridge, UK; Department of Psychology, University of Cambridge, Cambridge, UK; Institute of Public Health, University of Cambridge, Cambridge, UK; Institute of Psychiatry, Kings College London, London, UK; Department of Clinical Psychology, University of East Anglia, Norwich, UK

**Keywords:** Post traumatic stress disorder, Anxiety, Children, Trauma-focused cognitive behaviour therapy, PTSD

## Abstract

**Background:**

Following horrific or life-threatening events approximately 10 to 15% of young children develop post traumatic stress disorder (PTSD). The symptoms of this disorder are distressing - nightmares, flashbacks, anger outbursts and disturbed play. These symptoms cause major disruption to a child’s functioning and, if left untreated, can persist for many years. As yet, there are no established empirically-validated treatments for PTSD in young children. Trauma-focused cognitive behaviour therapy (TF-CBT) is a psychological intervention that is effective in treating the disorder in older children (8 to 12 years), adolescents and adults. This study examines TF-CBT adapted for children aged between 3 and 8 years.

**Methods/Design:**

This protocol describes a two-arm exploratory randomised controlled trial comparing TF-CBT to treatment as usual (TAU) in children aged 3 to 8 years with a principal diagnosis of PTSD following a single-event discrete trauma. Using a half-crossover design, 44 participants will be randomly allocated to receive the intervention or to receive TAU. Those allocated to TAU will be offered TF-CBT at the end of the ‘treatment’ period (approximately 12 weeks) if still indicated. The primary outcome is PTSD diagnosis according to DSM-5 criteria for children 6 years and younger at post-treatment. Secondary outcomes include effects on co-morbid diagnoses and changes in emotion and trauma symptoms at each of the follow-up points (post-treatment, 3-months, 12-months). Additionally, broader efficacy will be considered with regard to treatment feasibility, acceptability and service utilisation. The key targets of the intervention are trauma memory, the interpretation of the meaning of the event, and the management of symptoms.

**Discussion:**

This is the first European trial to examine the efficacy of TF-CBT in alleviating PTSD in very young children. As well as providing much-needed data on the utility of the intervention, this exploratory trial will also allow us to gather important information about the feasibility of delivering the treatment in UK National Health Service (NHS) settings, and its acceptability to the children and their families. This study will highlight aspects of the intervention that need improvement or modification in preparation for a full-scale evaluation in a larger sample.

**Trial registration:**

ISRCTN35018680, registered on 18 November 2013.

## Background

Post traumatic stress disorder (PTSD) is a deeply distressing and disabling anxiety disorder comprising symptoms of trauma re-experiencing (for example, flashbacks, nightmares), avoidance (for example, social withdrawal, emotional numbing), and hyper-arousal (for example, anger outbursts). Children, like adults, can develop PTSD following exposure to a number of discrete traumatic events, including interpersonal violence, road traffic collisions and burns [1-3]. Until the publication of the Diagnostic and statistical manual of mental disorders 5th edition (DSM-5: [4]) the diagnostic criteria had been adult-orientated, which led to an under-diagnosis of PTSD when compared to more developmentally sensitive criteria [5]. Young children have a limited ability to convey their subjective experiences, as a result of their limited cognitive and expressive language skills [6] and consequently it has been necessary to focus on more behavioural markers of distress. Young children typically display a different range of behaviours to adults, which Carpenter and Stacks summarised as including: refusal to eat or trouble keeping food down; extreme difficulty falling asleep or frequent night-waking; or changes in responsiveness to an adults efforts to soothe them that may include responding with heightened irritability, fearful expressions, crying or blank expressions under circumstances that do not normally produce these effects (that is face-to-face play or efforts to comfort) [7].

PTSD is frequently co-morbid with other psychiatric conditions such as anxiety and depression and markedly impairs educational, social and daily functioning [5]. Traumatic events are experienced by up to two thirds of children by age 16 [8] including in preschool and early school years, even when excluding abuse. A significant proportion (10 to 40%) of these younger children who are exposed to non-abuse traumas are severely affected and go on to develop PTSD [3,9-12]. For example, 10 to 14% of 3 to 8 year-old children are diagnosed with PTSD when assessed with developmentally appropriate criteria 6 months after presenting at a UK Emergency Department (ED) following acute trauma [3]. There is evidence that if left untreated, PTSD in children and young people can lead a chronic course lasting a number of years [13]. In support of this, 10 to 15% of 3 to 8 year-olds have previously been found to have PTSD 3 years after a trauma, which is in line with consensus in the field that chronic PTSD in young children following discrete traumas shows little spontaneous remission [3]. Part of the difficulty is that experiencing trauma at a young age can disrupt typical developmental processes, so higher levels of mood and behavioural problems are seen in comparison to control groups, increased difficulty coping with frustration, bouts of intense fear, sleep disturbances, regression in developmental achievements and social withdrawal [14], which can obviously lead to cumulative difficulties if left untreated.

The National Institute for Health and Care Excellence (NICE; [15]) guidelines recommend psychological treatment for PTSD, the 'gold standard' of which is trauma-focused cognitive behavioural therapy (TF-CBT). Importantly, the guidelines acknowledge that there is currently no evidence base for TF-CBT in younger children [15]. Despite clear clinical demand, the majority of children with PTSD go untreated. There is now an urgent need for the development of an evidence base for treatment of PTSD in young children. The current study meets this need.

Research evaluating TF-CBT in older children and adults has established an empirical base supporting the efficacy of the intervention [16]. Given the rapid development seen in younger children it is necessary to evaluate this paradigm separately to adults and older children. To date, there has only been one small pilot clinical trial examining TF-CBT in younger children, where children were allocated either to receive 12 sessions of manualised TF-CBT or to a waitlist control group [17]. The results of this study, conducted in New Orleans, USA, supported the feasibility and impact of developmentally-tailored CBT with a large effect size and treatment gains maintained at the 6-month follow-up. However, the results from this study are unlikely to be generalisable, particularly within the UK, given that the trial was conducted in an underprivileged, low social-economic group in an urban American setting [17]. Based on our previous work with older children and adolescents, aged 8 to 17 years, showing that TF-CBT improves symptoms of PTSD, anxiety, and depression, as compared to a waitlist control group, we adapted this programme [18] to provide a 12-session intervention suitable for young children (TF-CBT-YC) aged 3 to 8 years, following a single-incident traumatic event.

This study has been designed to answer three main questions. First, do children, diagnosed with PTSD, experience symptom reduction to sub-clinical levels following TF-CBT-YC? Secondly, is the treatment feasible and acceptable? Thirdly, is the cost of implementing TF-CBT-YC likely to represent a saving to the UK National Health Service (NHS) in terms of reducing the overall costs associated with service use post-trauma?

## Methods/Design

### Study design

**Figure 1:**
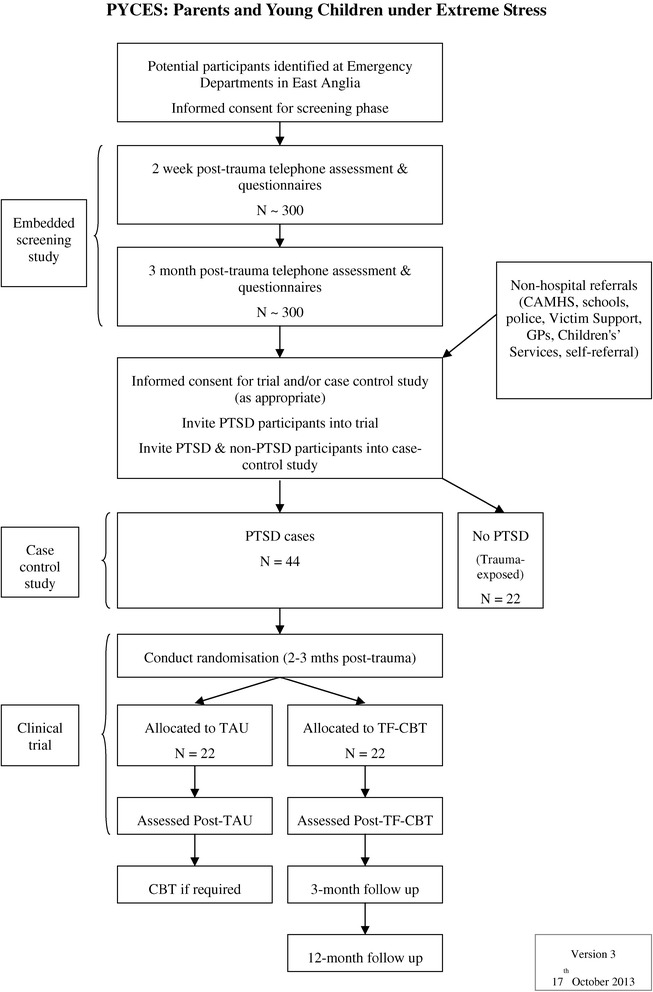
**Participant flow diagram.**

This study is a two-arm pilot randomised controlled trial (RCT) comparing TF-CBT-YC with treatment as usual (TAU). A half-crossover design will be employed such that participants allocated to the TAU arm will be offered the intervention after a 12-week waiting period if still indicated. Participants will be assessed 4 times during the study - at baseline, at post-TAU or -treatment, and at 3-month and 12-month follow-up for participants allocated to the treatment condition (see Figure 1). The post-treatment assessment will be conducted in the week following treatment completion and the post-TAU assessments will be at a comparable duration in the TAU arm.

### Participants

A total of 44 children aged 3 to 8 years with a principal diagnosis of PTSD will be randomised to either TAU (n = 22) or TF-CBT-YC (n = 22). For study inclusion, participants will meet criteria for a diagnosis of PTSD for children 6 years and younger according to the DSM-5 [4], as assessed using the Diagnostic Infant and Preschool Assessment (DIPA; [19]). Eligible participants will have experienced a discrete stressor (for example, accident, witnessing or experiencing violence, medical emergency or procedure). Children with both acute (1 to 6 months post-trauma) and chronic (>6 months post-trauma) PTSD will be included, given that there is evidence that TF-CBT-YC benefits both groups [17].

Exclusion criteria comprise head trauma (Glasgow Coma Score < 8); learning disability, but not specific learning difficulties; autism; another primary psychiatric diagnosis that warrants treatment using a psychological therapy ahead of the traumatic stress response; inability to speak English within the family; ongoing exposure to threat; and history of organic brain damage. Victims of chronic sexual or physical abuse will not be invited into the trial because of the need to involve specialist services. Appropriate referrals will be made in this case.

### Determination of sample size

Although a standard power calculation based on detecting treatment effects is the conventional approach to determining sample sizes for definitive clinical trials, the main aim of the current pilot trial is to investigate the core protocol and procedural uncertainties, in preparation for a later scaled up evaluation of the intervention in line with current guidance [20]. Nonetheless, we will obtain working knowledge of the likely effect size of the intervention itself. Seventeen participants per arm (TF-CBT-YC versus TAU) is enough to detect a difference in the recovery percentage as small as 44% on the DIPA diagnosis of PTSD, with 80% power at the 1-tailed 5% level of significance (45% in TAU versus 89% in TF-CBT-YC). These figures fall within the intent-to-treat outcomes in our previous trial in older children [18] (WL: 42% versus TF-CBT: 92%). These group sizes would also detect differences as small as 1.0 standard deviation (SD) unit on our continuous outcome of PTSD symptomatology, given the same power and level of significance. This would have provided sufficient power to detect group differences both in our previous trial [18] (adjusted effect size: Child PTSD Symptom Scale [21] = 2.48 SD units) and in the New Orleans trial [17] (adjusted effect size: PTSD symptom count = 1.32 SD units). To account for 20% potential attrition we will, therefore, recruit 22 young children per arm to the trial. It is important to note that the use of a half-crossover design with the initial TAU participants being offered TF-CBT-YC augments the sample size for the pre- to post-treatment comparisons on outcomes.

### Recruitment

There are two pathways through which participants will be recruited. The first is via an embedded prospective longitudinal study of trauma-exposed children, who have attended EDs in East Anglia, UK. Children attending EDs in hospitals in East Anglia after a single- event trauma (typically a road traffic accident or assault, but also including falls and other accidental or medical injuries) will be invited to take part in a screening study component of this project. Initially, parents of children exposed to a discrete trauma satisfying the study inclusion criteria will be sent details of the study by post and then contacted by a member of the ED team within 1 to 2 weeks post-trauma to assess eligibility and to obtain verbal consent to take part in this prospective longitudinal study. Consenting families will then be contacted at 2 to 4 weeks and again at 3 months post-trauma, at which point participants meeting a diagnosis of PTSD will be invited into the trial.

The second pathway through which participants will be recruited is via referral from community sources, including Child and Adolescent Mental Health Services (CAMHS), schools, Victim Support agencies, General Practitioners, the police (for example, Family Liaison Officers) and advertisements placed in local newspapers, on the Internet, in children’s centres and doctors surgeries.

### Participant allocation

**Figure 2:**
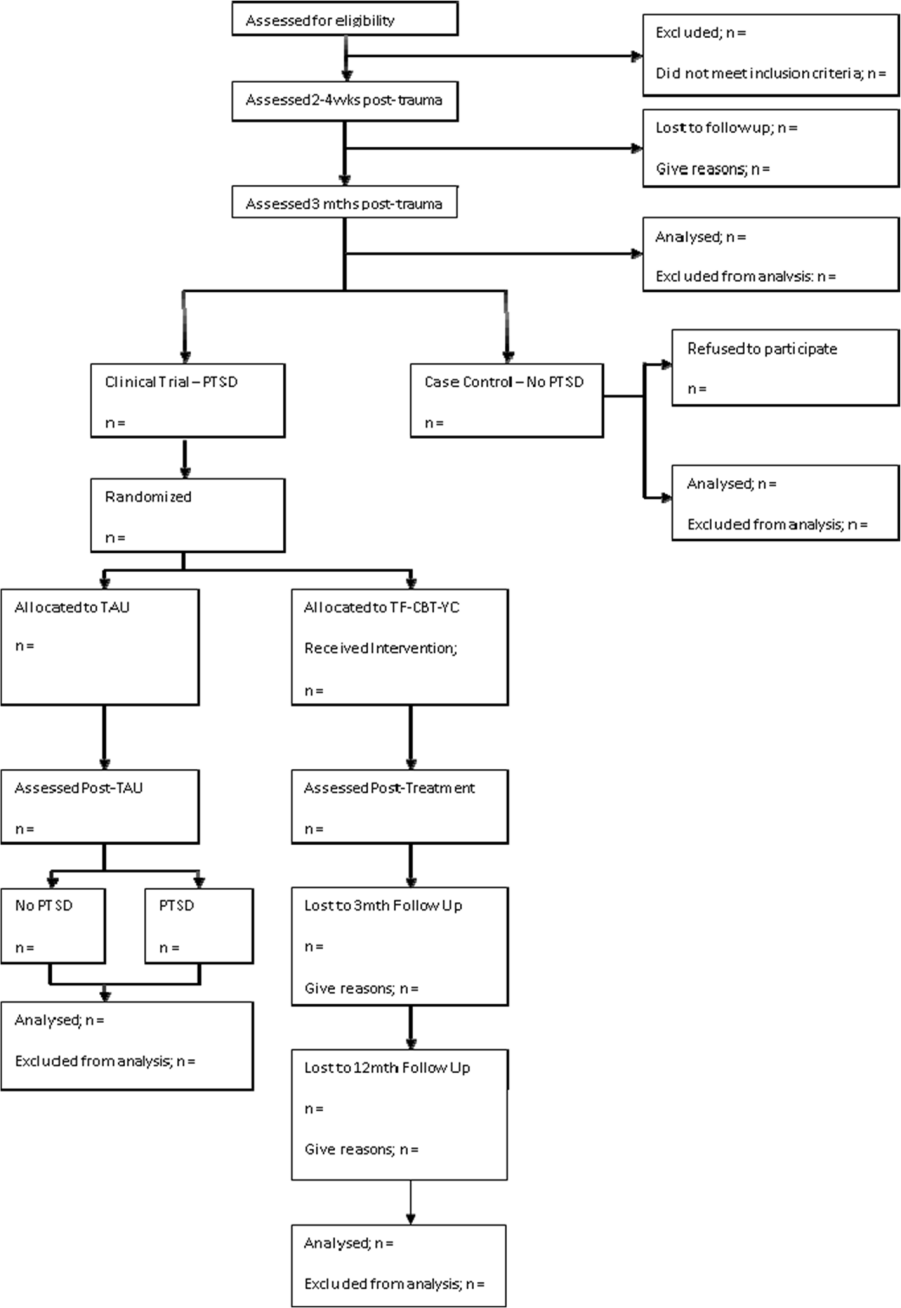
**Trial Consolidated Standards of Reporting Trials (CONSORT) diagram.**

Eligible participants will be randomised to TF-CBT-YC or TAU using a minimisation procedure suitable for small samples, constrained by age group (3 to 5 years or 6 to 8 years), gender, and initial clinical severity as assessed on the DIPA (high and low). These variables were selected because of their likely influence on treatment response. Randomisation will occur after consent has been taken and baseline measures have been completed. The trial team will Email the independent statistician (PW) details of the three stratifiers and, using the minimisation programme MinimPy [22], the statistician will randomise the participant and Email the results to the trial coordinator. Following randomisation, participants will be notified of their allocation by telephone, which is subsequently confirmed in writing. See Figure 2 for the Consolidated Standards of Reporting Trials (CONSORT) diagram.

### Interventions

TF-CBT-YC is a structured, manualised treatment, adapted from a treatment package developed for a previous trial of CBT for chronic PTSD (that is symptoms for > 6 months) in children and adolescents [18]. This treatment is based on a cognitive conceptualisation of PTSD [23,24] and specifically targets three maintaining factors of the disorder that are implicated in the aetiology of PTSD in youth: trauma memories, negative appraisals and maladaptive coping [23].

TF-CBT-YC is delivered to the child and parent or caregiver over 12 sessions of 60 to 90 minutes, supplemented by homework tasks. TF-CBT techniques for this age group have been derived both from our own published protocol for older children and adolescents developed for a previous trial [25], as well as the New Orleans protocol, used in the only published RCT for PTSD in this age group [17]. Treatment materials for this trial are developmentally sensitive with more complex cognitive exercises being introduced for the older children, as appropriate, in the 6 to 8 year age range. Specific techniques for the child and/or parent/caregiver include education about PTSD, recognition of feelings, training in coping skills, graduated exposure to the trauma through imagery, drawings and *in-vivo* work, the development of a coherent trauma narrative, the identification and reappraisal of erroneous trauma-related beliefs, and safety planning. Three sessions take place with the child and parent/caregiver together. The remaining sessions are divided into two halves. The first half involves the therapist and child only, but information is shared with the parent or caregiver via the therapist with the child’s knowledge. This facilitates parallel learning of the material and caregiver’s attunement to the child’s needs and feelings. The second half of each session involves just the therapist and parent or caregiver and provides a forum to discuss any problems, to assist the therapist in developing an understanding of the child’s behaviour outside of the session, and to plan and troubleshoot the homework assignments.

Participants in the TAU arm do not initially receive any TF-CBT from the trial team. However, they are able to continue any therapeutic input they have already during this period, and any for which they are newly referred, and if at post-treatment assessment the child still meets criteria for PTSD, they are then offered TF-CBT-YC, exactly as described above.

### Treatment integrity

Clinical psychologists will deliver the intervention. Fidelity and clinical adherence will be established through continued monitoring and independent rating. Specifically, clinicians will complete a modified version of the Therapist Fidelity Checklist (TFC; [26]), which is a session-by-session measure of compliance with the protocol, and these will be evaluated during weekly clinical supervision. In addition, treatment sessions will be video-taped and all assessments audio-taped. A random sample of 20% of recordings will be reviewed by the lead developer of TF-CBT (PS), who is not involved in day-to-day running of the trial.

### Measures

#### Primary clinical outcomes

The primary outcome will be recovery from PTSD using the DIPA at post-treatment. The DIPA is a psychometrically robust parental report semi-structured interview designed for this age group [19].

### Secondary clinical outcomes

As secondary outcomes, DIPA PTSD at 3-month and 12-month follow-ups, effects on co-morbid diagnoses (assessed with the DIPA) and changes on the parent-completed Young Child PTSD Checklist [26], the Pediatric Emotional Distress Scale (PEDS; [27]), and the Preschool Feelings Checklist (PFC: [28]), will be evaluated. These questionnaire measures provide continuous assessment of psychological symptoms of PTSD symptoms, general distress, and emotion functioning, respectively. Participants across both trial arms complete these questionnaires at baseline and post-treatment, with the trial arm also completing the measures at 3-months and 12-months post-treatment.

### Additional outcomes

Broader efficacy will be considered with regard to treatment outcome, treatment acceptability, and cost-effectiveness. Data to assess these additional outcomes will include qualitative, quantitative and health economics data as detailed below.

### Semi-structured interviews

Treatment outcome will be evaluated using quantitative measurement of potential PTSD symptom reduction from pre- and post-treatment as noted above, as well as an embedded qualitative study to elicit parents’ and children’s lived experiences of treatment. Semi-structured interviews will be conducted with a small sub-section (n ≈ 15) of those involved at different stages of the trial; specifically, treatment completers, treatment non-completers, treatment non-consenters and those who went from TAU to TF-CBT-YC. The aim is to garner the views of those who received treatment, either immediately, or after TAU. The views of those who did not consent or withdrew from the trial will also be sought to help us to understand the reasons for this. Interviews will cover topics that include: parents’ and children’s experience of the assessment process; treatment sessions; homework tasks; the practicalities of receiving treatment and perceived treatment impact. They will be conducted at 3 months post-treatment, or at 3 months following withdrawal from the study. It is acknowledged that this may be difficult in the case of those withdrawing or not consenting to be part of the study and, therefore, interviews may be conducted earlier as appropriate. Interviews will be conducted face-to-face, unless a participant declines, at which point a telephone interview will be offered.

### Additional quantitative measures

Feasibility and acceptability of the intervention will be assessed through a mixture of quantitative and qualitative methods. The feasibility and acceptability of TF-CBT-YC will be examined in three ways. First, a modified version of the clinician-completed Adaptability Checklist-Child (ACC16; [26]) will be used, which is a quantitative feasibility measure of the child’s ability to engage with and benefit from the different components of TF-CBT-YC (also used in the previous trial conducted in the USA, [17]). Second, treatment credibility items and the Therapeutic Alliance Scale [29,30] will be completed by parents or carers with their child’s input, at mid-treatment and post-treatment, to give an overview of the acceptability of the treatment to the child and their parents or carers. Third, a semi-structured interview as described above examines aspects of treatment feasibility and acceptability.

### Health economics measures

Data collection for the health economic evaluation will take a broad societal perspective, recording all hospital, community health and social services usage. Productivity losses resulting from time off work by parents or carers as a consequence of their child’s PTSD will be calculated using the human capital approach, a multiplication of days off work due to illness by the individual’s salary level [31]. Data will be collected using a structured questionnaire adapted for this study but based on the Child and Adolescent Service Use Schedule (CASUS), an instrument previously used in a trial of CBT for major depressive disorder in adolescents [32]. Data on TF-CBT-YC contact time and on indirect time (for example, supervision) spent delivering the intervention will also be collected. Cost information will be taken from national publications. Together, these data will allow us to test our resource utilisation instruments and permit preliminary estimates of TF-CBT-YC’s potential cost-effectiveness.

### Methodological aspects

#### Data collection

Quantitative outcome variable data for the trial will be collected at baseline, post-treatment, 3-month and 12-month follow-up. Qualitative interview data on treatment completers will be collected at 3-month follow-up, except in the case of participants who drop out earlier who will be followed-up as appropriate. Health economics data will be collected retrospectively (for the preceding 3-months) at all 4 assessment time points.

### Blinding

Outcome assessments are conducted by independent raters from The Cambridge Centre for Affective Disorders (C2:AD) who have no therapeutic relationship with the patients and are blind to treatment condition. The independent raters are psychology graduates, post-doctoral psychologists and clinical psychologists. All have received additional training specifically in the use of the outcome assessments, the opportunity to listen to conducted assessments and have direct feedback on their assessments through supervision. Further to this, a randomly selected 20% of recordings will be reviewed by the lead developer of TF-CBT (PS).

Double-blinding of patients and therapists is not possible due to the nature of the trial (that is a psychological intervention). Under no circumstance will unblinding of patients or therapists be possible because they are not blind to intervention allocation.

### Statistical analysis plan

Initial analyses of the quantitative primary and secondary outcome data will be conducted on an intent-to-treat basis, with subsequent analyses being per protocol (based on attendance at 50% of offered sessions), and carried out by the trial statistician (PW) following CONSORT standards [33]. Comparisons will be made across groups on all outcome measures. For the continuous scales, repeated measures analyses of variance (ANOVAs) will be used without adjustment in the first instance, and then adjusting for baseline levels of the relevant measures as covariates if indicated. For the caseness measure of PTSD, non-parametric analyses will be used (for example, Chi square/Fisher’s exact test).

Qualitative interview data will be analysed using thematic analysis [34], facilitated by NVivo 8 software (QSR International). For the health economic data, mean estimated treatment costs in the two trial arms will be compared using ANOVA and the robustness of the parametric tests confirmed using bootstrapping. The primary analysis will explore cost-effectiveness in terms of loss of PTSD diagnosis (that is 'recovery'). If a significant difference in the primary outcome is observed between the two treatment arms, cost-effectiveness will be explored through calculation of an incremental cost-effectiveness ratio and sensitivity analyses.

### Monitoring and data management

As a Phase I trial, a Data Management Committee was considered unnecessary and the trial team is, therefore, responsible for monitoring and data management. Data will be monitored for completeness, consistency, and plausibility using spot checks and plausibility checks carried out by the trial statistician. The trial lead, statistician, and trial coordinator will have full access to the final trial dataset. The study data will be reported in line with current CONSORT recommendations [33].

### Safety aspects

Adverse events refer to unwanted medical events (for example, worsening symptoms) occurring throughout the trial, regardless of whether they are causally related to the trial procedures. Adverse events are managed in line with UK Medical Research Council (MRC) guidelines [20,35,36] and, in the unlikely case of an adverse event, will be documented appropriately. Precautions have been taken to reduce the likelihood of adverse events occurring; for example, the interventions are delivered by clinical psychologists experienced in the management of risk and the treatment of PTSD and co-morbid disorders. In the case of any adverse events, participation in the trial will be discussed with the child and their parents to ensure the best outcome is achieved for the individuals involved. The trial is underwritten by the UK MRC in the case that any individual suffers harm or requires post-trial care.

### Ethical approval and protocol amendments

The project has received NHS ethics approval (Cambridge South Research Ethics Committee, MREC number 12/EE/0458) and local research governance approval has been obtained for all recruiting sites; Table 1 includes a list of sites and the approvals in place. The study personnel, a Trial Management Group and a Trial Steering Committee will ensure that the study is conducted within appropriate NHS and professional ethical guidelines. Good Clinical Practice training will have been undertaken by all those directly involved in running the study. Full informed consent will have been obtained from all participating families. Protocol amendments will be circulated to the board of ethics, research and development, and trial team. Relevant adjustments will be made to any published protocol.

**Table 1:** **List of recruitment sites and approvals**

**Recruitment site**	Site-Specific Information (SSI)*	Participant Identification Centre (PIC)*
MRC CBU	✓	
HSB	✓	
CPFT	✓	
CUH	✓	
West Suffolk Hospital	✓	
Cambridgeshire PCT		✓
Bedfordshire PCT		✓
West Essex PCT		✓
Norfolk PCT		✓
Peterborough Hospital		✓
Cambridgeshire Community Services		✓

*Whether a site had an SSI or PIC was guided by local Research and Development preference and the type of recruitment carried out at that site.

PCT, Primary Care Trust.

### Confidentiality

All participants will give written informed consent prior to being assessed for eligibility to be included in any part of the study. To maintain confidentiality, all participants are given a trial number so that personally identifiable information is not linked to assessment or trial information.

### Dissemination policy

There are no publication restrictions and findings will be disseminated broadly to participants, healthcare professionals, the public, and other relevant groups.

## Discussion

PTSD is a distressing and debilitating mental health condition. If left undiagnosed and untreated, PTSD can take a chronic course and lead to significant functional impairment [13]. TF-CBT is an evidence-based treatment with demonstrated efficacy for older children, adolescents and adults [25]. However, there is no evidence base for its efficacy in younger children. There has been a growing recognition of the need to develop an age appropriate treatment model for younger children; this exploratory RCT meets this objective. The timely nature of this trial is underscored by the inclusion of a preschool age criteria included in the recent DSM-5, reflecting the growing recognition that PTSD in this age group needs to be addressed.

Data evaluating the effectiveness of TF-CBT-YC will provide an initial step towards providing an empirical base for the treatment of young children with PTSD. If superiority to TAU can be demonstrated, the results from this trial will support the need for a fully powered definitive trial to examine TF-CBT-YC as the treatment of choice for young children with PTSD. Not only is this trial needed to examine efficacy, feasibility and acceptability, but the economic evaluation of this treatment will determine its cost-effectiveness over the medium to long term and, therefore, TF-CBT-YC’s applicability to delivery through NHS channels. Accordingly, TF-CBT-YC has the potential to bring benefits to young children affected by PTSD, but also to the health system more broadly.

## Trial status

Recruitment started in July 2013.

## Abbreviations

ACC: Adaptability Checklist-Child
ANOVA: analysis of variance
C2:AD: The Cambridge Centre for Affective Disorders
CAMHS: Child and Adolescent Mental Health Services
CASUS: Child and Adolescent Service Use Schedule
CONSORT: Consolidating Standards of Reporting Trials
CPFT: Cambridgeshire and Peterborough NHS foundation trust
CUHFT: Cambridge University Hospitals NHS Foundation Trust
DIPA: Diagnostic Infant and Preschool Assessment
DSM-5: Diagnostic and Statistical Manual of Mental Disorders - 5^th^ Edition
ED: Emergency Department
(MRC) CBSU: (Medical Research Council) Cognition and Brain Sciences Unit
NHS: National Health Service
NICE: National Institute for Health and Care Excellence
PEDS: Pediatric Emotional Distress Scale
PFC: Preschool Feelings Checklist
PTSD: post traumatic stress disorder
RCT: randomised controlled trial
REC: Research Ethics Committee
TAU: treatment as usual
TFC: Therapist Fidelity Checklist
TF-CBT: trauma-focused cognitive behaviour therapy
TF-CBT-YC: TF-CBT-young children
